# Effect of a 90-Minute Nap at Different Times of the Day on Physical Performance, Psycho-Cognitive Responses, and Perceived Recovery in Trained Youth Male Athletes

**DOI:** 10.3390/sports13110395

**Published:** 2025-11-06

**Authors:** Arwa Jebabli, Slaheddine Delleli, Nourhène Mahdi, Khouloud Ben Maaoui, Juan Del Coso, Hamdi Chtourou, Luca Paolo Ardigò, Ibrahim Ouergui

**Affiliations:** 1 High Institute of Sport and Physical Education of Sfax, University of Sfax, Sfax 3000, Tunisia; jebabliarwa@gmail.com (A.J.); sdelleli2018@gmail.com (S.D.); nourhene648@gmail.com (N.M.); benmaaouikhouloud88@gmail.com (K.B.M.); h_chtourou@yahoo.fr (H.C.); 2 Research Laboratory, Education, Motricity, Sport and Health, EM2S, LR19JS01, University of Sfax, Sfax 3000, Tunisia; 3 Physical Activity, Sport and Health, Research Unit, UR18JS01, National Sport Observatory, Tunis 1003, Tunisia; 4 Centre for Sport Studies, Rey Juan Carlos University, 28943 Fuenlabrada, Spain; juan.delcoso@urjc.es; 5 Department of Teacher Education, NLA University College, 0166 Oslo, Norway; 6 High Institute of Sport and Physical Education of Kef, University of Jendouba, Kef 7100, Tunisia; 7 Research Unit: Sport Sciences, Health and Movement, UR22JS01, University of Jendouba, Kef 7100, Tunisia

**Keywords:** repeated sprint, fatigue, sleep, athletic performance

## Abstract

Napping is recognized as a strategy to enhance athletic performance. However, the optimal timing and duration for maximizing its benefits remain unclear. This study investigated the effects of a 90 min nap at different times on physical performance, psycho-cognitive responses, and perceived recovery in trained youth male athletes. Fourteen athletes (18 ± 1 years) completed four conditions in a randomized crossover design: (1) No-nap-13h, (2) No-nap-15h, (3) Nap-13h, and (4) Nap-15h. After each condition, athletes performed a 5 m shuttle run test (5mSRT) and were assessed on best distance (BD), total distance (TD), and fatigue index (FI). Ratings of perceived exertion (RPE) were recorded after each 5mSRT repetition, whereas muscle soreness (DOMS) and recovery (PRS) were assessed post-test and 24 h later. The digit cancelation test (DCT), feeling scale (FS), Stanford Sleepiness Scale (SSS), and Hooper Questionnaire evaluated sleep quality and psycho-cognitive state. Results showed that the athletes felt greater sleepiness before Nap-15h and after Nap-13h versus the no-nap conditions. TD was higher in Nap-13h than Nap-15h (*p* = 0.001) and No-nap-15h (*p* = 0.0009). BD was higher in Nap-13h versus No-nap-15h and No-nap-13h, while RPE was higher in Nap-13h versus No-nap-13 h, Nap-15h, and No-nap-15h (all, *p* < 0.05). DCT scores were also higher in Nap-13h. No significant effects were found for FI, FS, or Hooper. In conclusion, a 90 min nap at 13:00 was more effective than a later nap or no nap in improving performance and recovery, suggesting benefits for afternoon training or competitions.

## 1. Introduction

Sleep is a complex physiological process regulated by various brain regions through the modulation of multiple neurochemical systems, particularly neurotransmitters and neuropeptides [1]. Evidence suggests that sleep during rest periods plays a crucial role in the body’s repair processes, supporting the homeostatic regulation of the autonomic, neuroendocrine, and immune systems [2]. Sufficient sleep is fundamental for all individuals, but it is particularly crucial for athletes, as it supports physical recovery between training sessions and after competitions. Additionally, sleep quality plays a vital role in enhancing athletic performance, optimizing cognitive function, and reducing injury risk during sports competitions [3]. In this context, athletes frequently face multiple sources of stress, such as intensive training programs, early sessions, altitude exposure, competitive pressure, and frequent travel, which can disrupt sleep and pose challenges to sustaining performance and recovery [4]. Therefore, identifying effective strategies to enhance recovery and mitigate sleep disturbances caused by these stressful situations may be crucial for optimizing athletic performance, maintaining physical and mental well-being, and reducing the risk of fatigue-related concerns.

Given its essential role in recovery and performance, sleep is now recognized as a key component of sports training programs [5,6]. Research indicates that insufficient sleep can alter glucose metabolism, reduce cognitive performance, increase perceived exertion, and compromise reaction time—all critical factors for athletes [5]. In addition, chronic sleep restriction can lead to hormonal imbalances, increased risk of overtraining, and decreased immune defenses, making athletes more vulnerable to illness and injury [7]. It is therefore essential to prioritize sleep hygiene and recovery strategies to optimize physical and mental preparation in sport.

Human performance often declines during the post-lunch dip period, usually occurring between 13:00 h and 16:00 h, [7]. This decline is attributed to heightened sleepiness, decreased vigilance and cognitive alertness [8]. In order to compensate for the decreased performance that results from the post-lunch dip, daytime napping is considered a recovery strategy [9]. Given that nocturnal sleep should be 7–9 h in duration and of good quality [10], daytime napping could be used as a prophylactic strategy complementing a full night’s sleep to enhance athletic performance [11,12]. Because napping has several benefits, particularly for recovery and improving physical performance, sports science researchers are interested in using naps as performance/recovery tools [8]. Napping has also been positively associated with positive mood [13], psychomotor responses [14], and cognitive performance [3].

Recent evidence indicates that napping, in addition to offsetting the effects of partial sleep loss from the prior night, can offer further advantages even after adequate nocturnal sleep [15]. For instance, in well-rested subjects, Chtourou et al. [8] indicated that a 45 min daytime nap taken at 14:00 or 15:00 significantly enhanced performance in a 5mSRT conducted 30 min post nap (*p* < 0.05). Supporting this, Boukhris et al. [16] found that a 25 min nap at 13:30 led to improved sprint performance (*p* < 0.01) and reduced fatigue (*p* < 0.05), while Hsouna et al. [3] reported that both short (20 min) and medium (60 min) naps at 14:00 enhanced repeated sprint ability in semi-professional soccer players (*p* < 0.05), with the longer nap offering greater benefits. Under sleep-restricted conditions, Romdhani et al. [17] observed notable improvements in cognitive (*p* < 0.01) and physical performance (*p* < 0.05) following a 90 min nap at 13:00, indicating the potential advantages of longer naps. However, the potential benefits of napping were not always documented. In fact, Botonis et al. [9] and Petit et al. [18] reported that 20 min naps did not significantly influence either short-duration physical exercise or cognitive outcomes (*p* > 0.05). The inconsistencies in studies’ findings regarding the benefits of napping can likely be attributed to variations in nap duration and the time of day at which a nap was taken [9]. Regarding nap duration, while some reports showed that a longer duration yielded a more reliable performance enhancement [14,16,17,19], other studies have demonstrated that the benefits of longer nap durations are diminished by negative outcomes of sleep inertia or disrupted subsequent nighttime sleep, and in some instances the benefits may be reversed [20,21]. From another angle, the recuperative effect of a nap depends on its timing relative to the 24 h circadian cycle [1]. Highlighting the importance of timing, Abdessalem et al. [22] found that naps taken at 14:00 and 15:00 resulted in better performance outcomes compared to those taken at 13:00.

While the aforementioned studies portray the important effects of nap opportunities on performance and underscore that both the duration and timing of naps are pivotal for optimizing physical performance, how increasing nap duration affects performance across different timepoints remains to be established. Therefore, the present study investigated the effect of 90 min daytime (i.e., 13:001 and15:00) napping on physical performance, recovery perception and psycho-cognitive responses in trained youth male athletes. Given that napping was associated with enhanced alertness, reaction time, mood, and several aspects of physical performance [9,15], it was hypothesized that napping at either time of day would enhance performance compared to no-nap conditions. Taking into consideration that a nap at 13:00 would coincide with the post-lunch dip [8], we hypothesized that greater improvements in performance-related outcomes will be recorded at this time of day compared to other conditions.

## 2. Materials and Methods

### 2.1. Participants

**Figure 1:**
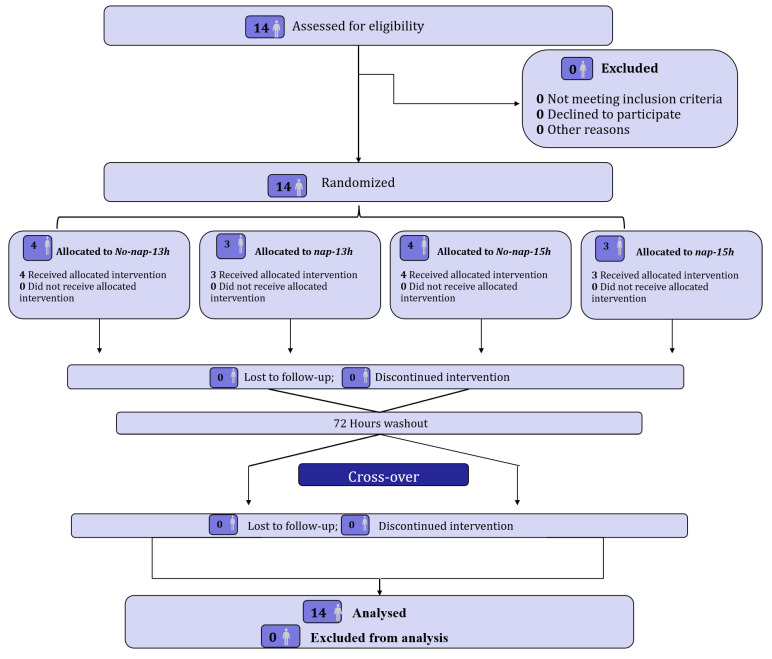
Participants’ flow diagram.

The required sample size was calculated a priori using G*power software (version 3.1.9.4, Université de Kiel, Kiel, Germany). The within-factors ANOVA with repeated measures test was used with α set at 0.05, power at 0.80, and a predicted partial eta squared of 0.52, which were determined from a previous study [19] assessing the effect of 90 min naps on 5mSRT performance. The power analysis revealed that a minimum sample size of 12 participants was required to reach an actual power of 82%. During data screening, several outcome variables deviated from normality. Accordingly, we prespecified a rank-based, nonparametric primary analysis for those outcomes (see Statistical Analysis Section). To preserve the targeted power when using nonparametric tests, we adjusted the parametric sample-size estimate using the asymptotic relative efficiency (ARE), which quantifies the efficiency of the rank-based test relative to its parametric counterpart under normal theory. Specifically, for repeated measures with k conditions, we used ARE = 0.955 × k/(k + 1) and computed the required nonparametric sample size as n_np_ = n_p_/ARE. Because ARE < 1 under normality, this constitutes a conservative inflation of the parametric estimate to maintain power for the nonparametric analysis [23]. The calculation revealed that 10 participants were required under this new calculation; however, taking into consideration the risk of drop out during the study, 14 trained youth male athletes (mean ± SD; age: 18 ± 1 years; height: 1.7 ± 0.09; body mass: 60.35 ± 5.83 kg) were recruited as volunteers to participate in the present study. The participants were regional team sports athletes from local clubs who engaged in high-intensity intermittent exercise >2–3 days per week for an average of 2 h per session with training experience of 5 ± 1 years. To be eligible, participants should fulfill the following inclusion criteria: (i) absence of any pathological sleep disorders, i.e., Pittsburgh Sleep Quality Index score < 5 [24], (ii) nonsmoking status and no consumption of alcohol drinks, (iii) not suffering from any injury in the three months prior to experimental testing, (iv) engaged in structured exercise training including high-intermittent exercise training. Participants maintained their habitual training routines throughout the experiment period. In the month preceding the experiment, participants completed daily sleep diaries documenting time in bed, sleep latency, bedtime and wake time, and wake after sleep onset. According to the Pittsburgh Sleep Quality Index, no sleep disturbances were reported [24]. Participants and their parents were provided with a thorough explanation of the protocol prior to participating in the study, after which an agreement was granted and written informed consent was signed by participants and/or parents or legal guardians. The present study was conducted according to the Declaration of Helsinki for human experimentation, and the protocol was fully approved by the Local Research Ethics Committee [Comité de Protection des Personnes SUD (CPP SUD) N° 0412/2022; approval date: 11 May 2022]. The flow diagram of participants’ recruitment and participation is shown in Figure 1.

### 2.2. Experimental Design

**Figure 2:**
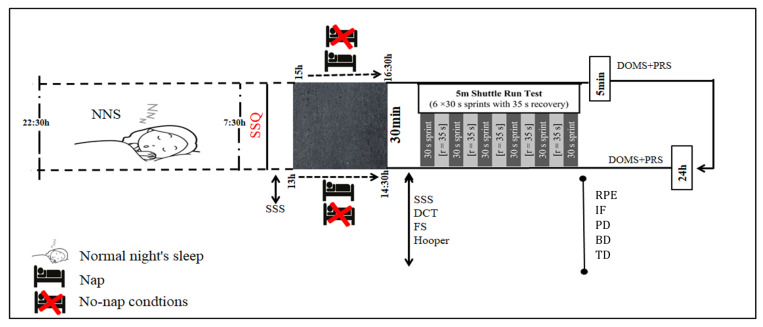
Schematic representation of the study protocol. DOMS: Delayed onset of muscle soreness, RPE: rating of perceived exertion, PRS: perceived recovery status, DCT: digit cancelation test, SSQ: Subjective Sleep Quality, SSS: Stanford Sleepiness Scale, FS: feeling scale, Hooper: Hooper questionnaire, BD: best distance, TD: total distance, PD: percentage decrement, IF: fatigue index.

Following the Consolidated Standards of Reporting Trials (CONSORT) guidelines this study employed a randomized crossover design [25] (Supplementary Table S1) to investigate the effects of napping on high-intensity athletic performance. One week prior to testing, athletes submitted to a familiarization session with the experimental procedures. Afterward, participants were enrolled in an experiment comprising four conditions identical in all respects except for the presence of a nap and its timing These sessions resulted from the combination between two conditions (no napping vs. 90 min of napping) and two measurement times (i.e., 13:00 and 15:00) and were as follows: (1) No-nap-13h, (2) No-nap-15h, (3) Nap-13h, and (4) Nap-15h. A block randomization method was used using an Excel spreadsheet (Microsoft Excel 2007, Redmond, WA, USA) to ensure a balance with respect to the sample size. Each participant was randomly allocated to the different conditions with a crossover design and a resting period of 72 h was applied between sessions [16,19,26]. The experimental sessions were conducted following a normal night’s sleep of 9 h, which was confirmed by athletes completing the Subjective Sleep Quality (SSQ) questionnaire before the start of each session [27]. In the sessions with napping, and after a period of adaptation to their sleep environment, participants were invited to take a nap of 90 min starting at 13:00 or at 13:00, after which a 30 min period was allowed for athletes to overcome any residual sleep [28]. The 90 min nap duration was chosen to enable participants to complete a full sleep cycle, including both slow-wave and rapid eye movement (REM) sleep [3]. In trials without napping, participants remained seated, awake, and at rest in a dark, quiet sleep room; compliance was confirmed by the researchers. In order to control for the effect of food intake on sleepiness and performance, all participants were instructed to consume a standardized isocaloric lunch approximately 90 to 120 min prior to each trial. This timing was chosen to align with the natural post-lunch dip window while avoiding acute postprandial effects that might interfere with nap quality or performance measures. The standardized isocaloric lunch contained 55% of carbohydrates, 20% protein, and 25% fats. The meal was specifically designed to ensure a stable energy level, thereby preventing any interference with the nap [29]. To avoid any stimulating effects, such as those from caffeine, participants were instructed to abstain from all caffeine-containing foods and beverages for at least 24 h before each experimental session. The Stanford Sleepiness Scale (SSS) was administered just before the nap and again after the 90 min nap period (i.e., at approximately 14:30 and 16:30, respectively) to evaluate athletes’ alertness [21]. Additionally, immediately after napping in the nap conditions or after resting in the no-nap conditions, participants were asked to rate their general emotional valence using the feeling scale (FS) [30]. Then, they performed the digit cancelation test (DCT) to assess their attention and alertness [31] and the Hooper Questionnaire to monitor their recovery status [32]. For the two no-nap conditions and the two nap opportunity conditions, participants spent the remaining time up to the onset of the exercise protocol (around 20 min) reading books, watching videos on television, or playing video games in a comfortable armchair [3]. Just 30 min post napping, participants completed 10 min of standardized warm up consisting of light runs and dynamic stretching. Then, they performed the 5 m shuttle run test (5mSRT) following previous recommendations [33]. The rating of perceived exertion (RPE) was collected after each repetition of the 5mSRT using the Borg CR10 scale [34]. Additionally, the PRS and DOMS scales were completed 5 min and 24 h after the 5mSRT [35]. A depiction of the experimental design is presented in Figure 2.

### 2.3. Data Collection and Analysis

#### 2.3.1. 5 m Shuttle Run Test

The 5mSRT is a high-intensity, intermittent sprint test used to assess anaerobic capacity, agility, and repeated sprint ability. It involves short, maximum effort sprints with frequent changes in direction, simulating the demands of many team sports. As described by Boddington et al. [33], the protocol includes six repetitions of 30 s shuttle sprints. In each repetition, the athlete sprints back and forth over a fixed shuttle distance, 5 m, 10 m, 15 m, 20 m, etc., with the distance increasing from one repetition to the next. Each 30 s sprint (i.e., one shuttle) is followed by a 35 s passive recovery period. The test is conducted on a flat surface with two parallel lines marked 5 m apart. For each repetition, the athlete sprints 5 m to the opposite line, touches it with one foot, executing a sharp 180-degree turn, and sprints back to the starting line. The distance covered during each repetition and the greatest distance [best distance (BD)] during a 30 s shuttle, as well as the total distance (TD) covered during the six 30 s shuttles, were recorded. Additionally, the fatigue index (FI) (Equation (1)) and percentage decrement (PD) (Equation (2)) [19,33] were calculated as follows:

- Total distance (TD) (m) = sum of distances covered during the 6 × 30 s shuttles;
- Best distance (BD) (m) = highest distance covered during one of the 6 × 30 s shuttles;
- Fatigue index (FI) (%):

FI (%) = [(shuttle 1 + shuttle 2)/2 − (shuttle 5 + shuttle 6)/2]/((shuttle 1 + shuttle 2)/2) × 100(1)

- Percentage decrement (PD) (%):

PD (%) = [(BD × number of sprints) − TD]/((BD × number of sprints)) × 100(2)

#### 2.3.2. Stanford Sleepiness Scale

The SSS is a visual analog scale measuring subjective sleepiness, alertness, and sleep quality. Sleepiness is assessed on a scale of 1 to 7, where 1 means “very active” and 7 means “very tired”. Alertness measure ranged from 0 to 10, where 0 means “extreme sleepiness” and 10 means “extreme alertness”. Subjective assessment of sleep quality is based on a scale of 0 to 10, where 0 means “no sleep”, 5 means “some sleep is interrupted”, and 10 means “deep, uninterrupted sleep” [36].

#### 2.3.3. Feelings Scale

The FS assesses general emotional valence, indicating levels of pleasure and dissatisfaction, following the methodology of Hardy & Rejeski [30]. It is a 10-point bipolar scale ranging from +5 to −5, with verbal associations of very good (+5), good (+3), moderately good (+1), neutral (0), bad (−1), bad (−3), and very bad (−5) [30].

#### 2.3.4. The Hooper Questionnaire

The Hooper questionnaire provides a subjective evaluation of sleep quality, fatigue, stress and muscle soreness on a scale ranging from 1, “very, very low”, to 7, “very, very high”, for fatigue, stress, and muscle soreness and from 1, “very, very good”, to 7, “very, very bad”, for sleep. In this study, the sleep scale refers to the participant’s “level of sleepiness at the time of the test” [32]. The Hooper index score was then calculated as the sum of the score of these four items [32].

#### 2.3.5. The Digit Cancelation Test

The DCT is a neuropsychological assessment tool used to evaluate attention, cognitive processing speed, and executive functioning. It assesses an individual’s ability to focus, process information, and sustain attention for extended periods. It consists of four pages, containing 600 numbers of 1 to 5 digits spread over 36 lines [31]. It was developed to assess various aspects of prefrontal cortex performance, such as attention, information processing speed, and executive functioning [31]. Participants had to erase as many three-digit numbers as possible for 1 min. The number of targets was 187, with 2 to 8 three-digit numbers randomly distributed per line, separated by periods, with a space on either side. The total number of targets correctly erased represents an assessment of an athlete’s vigilance [31].

#### 2.3.6. Rating of Perceived Exertion

The RPE uses a scale from “0” to “10” with verbal descriptors, reflecting the increasing intensity of perceived exertion: (0 = nothing at all; 1 = very weak; 2 = weak; 3–4 = moderate; 5–6 = strong; 7–9 = very strong; and 10 = extremely strong) [34].

#### 2.3.7. The Delayed Onset Muscle Soreness Scale

The DOMS scale is a subjective measure for muscle pain in the lower limbs, ranging from “1” (no pain), to “5”, to “10” (very painful) [37].

#### 2.3.8. Perceived Recovery Status Scale

The PRS evaluates athletes’ perceived recovery. All participants completed a self-assessment report in the form of an 11-point representation scale from 0, “very little recovery/extremely tired”, to 10, “very good recovery/very energetic” [35].

### 2.4. Statistical Analyses

Statistical analysis was performed using STATISTICA version 10 software (StatSoft, Paris, France). The normality of the dataset was checked using the Shapiro–Wilk test. Data were presented as mean and standard deviation (SD) for RPE and DCT and as median and interquartile range for the other variables, which presented non-normal distribution data. Comparisons among conditions were performed using a one-way repeated measures ANOVA for RPE and DCT and the Friedman test for the other outcomes. When appropriate, post hoc pairwise comparisons were conducted using Bonferroni tests for RPE and DCT values and Wilcoxon signed-rank test for the other variables. For RPE and DCT, the magnitude effect was determined using Cohen’s d and the 95% confidence interval of the difference (95%CI_d_) was calculated. The magnitude of the differences was classified according to Hopkins [38]: ≤0.20 (very small); ≤0.60 (small); ≤1.20 (moderate); ≤2.0 (large); ≤4.0 (very large); and >4.0 (extremely large). For the other variables, the rank biserial correlation coefficient (r) was calculated using the Wilcoxon z-scores and the total number of observations (N) (i.e., r = Z/√N) and considered as 0.1 to <0.3 (small), 0.3 to <0.5 (moderate), and ≥0.5 (large) [39]. A significant difference at the level of *p* < 0.05 was accepted for all analyses.

## 3. Results

### 3.1. Physical Performance

#### 3.1.1. Total Distance

Statistical analysis revealed a significant effect of the experimental conditions under testing (Chi2 = 34.89, N = 14; df = 3; *p* < 0.001). The pairwise comparisons revealed that TD was higher in Nap-13h compared with the No-nap-13h (*p* < 0.001; r = 0.88 (large)), Nap-15h (*p* = 0.013; r = 0.66 (large)), and No-nap-15h conditions (*p* < 0.001; r = 0.88 (large)) (Table 1).

#### 3.1.2. Best Distance

The Friedman test showed a significant effect of the experimental conditions under testing (Chi2 = 22.64, N = 14; df = 3; *p* < 0.001). Pairwise comparisons revealed that BD was higher in the Nap-13h group compared with No-nap-15h (*p* = 0.002; r = 0.82 (large)) and No-nap-13h (*p* = 0.006; r = 0.72 (large)) (Table 1).

#### 3.1.3. Fatigue Index

The Friedman test showed that there was no effect of the experimental conditions under testing (Chi2 = 6.07, N = 14; df = 3; *p* = 0.11) for FI (Table 1).

#### 3.1.4. Percentage Decrement

The Friedman test showed that there was no effect of the experimental conditions under testing (Chi2 = 7.35, N = 14; df = 3; *p* = 0.06) for PD (Table 1).

### 3.2. Rating of Perceived Exertion

There was a main effect of condition for RPE (F_(3, 39)_ = 8.21; ηp^2^ = 0.39; *p* = 0.0002). RPE values were higher after Nap-13h than Nap-15h (95%CI_d_ = 0.30 to 2.32; d = 1.663; *p* = 0.005), No-nap-13h (95%CI_d_ = 0.26 to 2.28; d = 1.056; *p* = 0.007), and No-nap-15h (95%CI_d_ = 0.68 to 2.70, d = 1.931, *p* < 0.001; Table 1).

### 3.3. Sleep Quality

**Figure 3:**
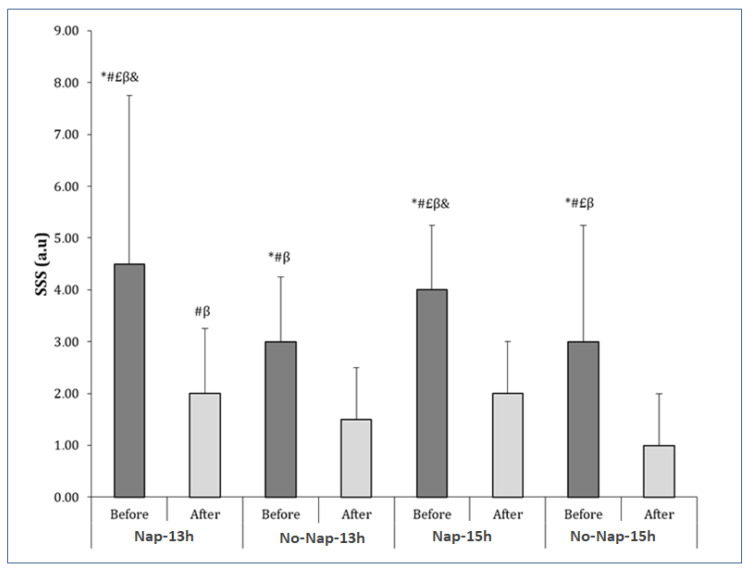
Stanford Sleepiness Scale (SSS) values are presented as medians (interquartile range) before and after nap. *: significantly different from after nap 13 h; ^#^: significantly different from after control 13 h; ^£^: significantly different from after nap 15 h; ^β^: significantly different from after control 15 h; ^&^: significantly different from before control 15 h; a.u.: arbitrary unit.

The Friedman test showed a significant effect of the experimental conditions under testing (Chi2 = 45.81, N = 14; df = 7; *p* < 0.001). Higher sleepiness was noted before Nap-13h than after Nap-13h (*p* = 0.0035; r = 0.78 (large)), after No-nap-13h (*p* = 0.0037; r = 0.77 (large)), after Nap-15h (*p* = 0.003; r = 0.79 (large)), before No-nap-15h (*p* = 0.02; r = 0.61 (large)) and after No-nap-15h (*p* = 0.002; r = 0.81 (large)). Moreover, sleepiness before Nap-15h was higher than after Nap-13h (*p* = 0.005; r = 0.76 (large)), after No-nap-13h (*p* = 0.001; r = −0.85 (large)), after Nap-15h (*p* = 0.001; r = 0.85 (large)), before No-nap-15h (r = 0.57; *p* = 0.03), and after No-nap-15h (*p* = 0.0009; r = 0.88 (large)). In addition, after Nap-13h, higher sleepiness values were noted than after No-nap-13h (*p* = 0.04; r = 0.54 (large)) and No-nap-15h (r = 0.78; *p* = 0.004). However, after No-nap-15h, sleepiness was lower than before No-nap-13h (*p* = 0.004; r = 0.78 (large)) and before No-nap 15h (*p* = 0.007; r = 0.007 (large)), Figure 3).

### 3.4. Psycho-Cognitive Parameters

#### 3.4.1. The DCT

There was a main effect of condition for DCT (F_(3, 39)_ = 13.96; ηp^2^ = 0.52; *p* < 0.001). Higher DCT values were recorded after Nap-13h than No-nap-13h (95%CI_d_ = 9.95 to 26.05; d = 2.205; *p* < 0.001), Nap-15h (95%CI_d_ = 1.80 to 17.91; d = 1.421; *p* = 0.009), and No-nap-15h (95%CI_d_ = 5.44 to 21.55; d = 1.733; *p* < 0.001). Moreover, Nap-15h elicited higher values than No-nap-13h (95%CI_d_ = 0.09 to 16.20; d = 0.981; *p* = 0.04, Table 2).

#### 3.4.2. Feeling Scale

For FS score, there was no significant effect of the experimental conditions under testing (Chi2 = 2.03, N = 14; df = 3; *p* = 0.57) (Table 2).

**Table 2:** Representation of the results of the feeling scale (FS), digit cancelation test (DCT) recorded after each testing session.

	Nap-13h	No-Nap-13h	Nap-15h	No-Nap-15h
DCT (a.u.)	72.57/6.77 *^,&,$^	54.57/9.35	62.71/7.10 ^&^	59.07/8.69
FS (a.u.)	1.00 (3)	0.50 (2.25)	1.00 (2.25)	1.00 (2.25)

Values are presented as medians (interquartile range) and means/standard deviations, depending on the distribution of each variable. *: significantly different compared to No-nap-15h; ^&^: significantly different compared to No-nap-13h; ^$^: significantly different to Nap-15h; a.u.: arbitrary unit.

#### 3.4.3. The Hooper Questionnaire

**Figure 4:**
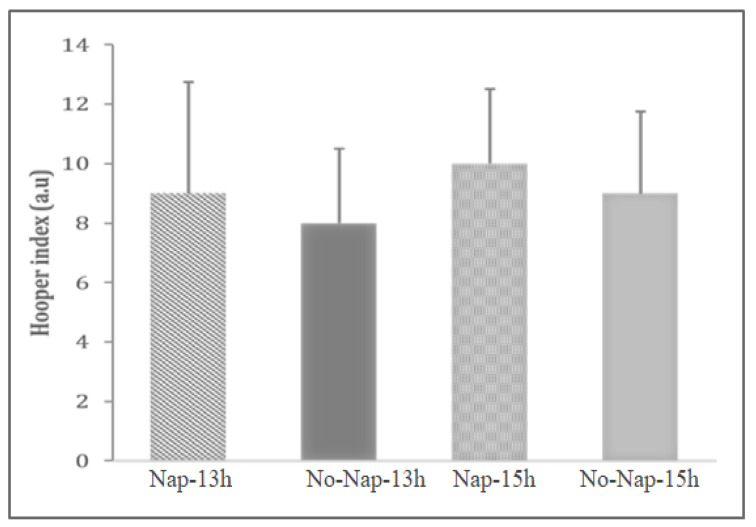
Hooper index recorded during each testing session. Values are presented as medians (interquartile range), a.u.: arbitrary unit.

The statistical analysis showed no significant main effects of condition on Hooper index (F_(3, 39)_ = 1.17; ηp^2^ = 0.08; *p* = 0.33, Figure 4).

### 3.5. Perceived Recovery

#### 3.5.1. Delayed Onset of Muscle Soreness

The statistical analysis of DOMS showed a significant effect of the experimental conditions under testing (Chi2 = 14.44, N = 14; df = 7; *p* = 0.04). DOMS scores were higher 24 h than 5 min after (*p* = 0.01; r = 0.63 (large)) 5mSRT only under the No–nap-15h condition (Table 3).

#### 3.5.2. Perceived Recovery Status

Statistical analysis showed a significant effect of the experimental conditions on PRS (Chi2 = 23.54, N = 14; df = 7; *p* = 0.0014). At 5 min, PRS was significantly higher in the No-nap-15h condition compared to 5 min post Nap-13h (*p* = 0.018, r = 0.63 (large)) and 5 min post Nap-13h (*p* = 0.008; r = 0.71 (large)). In addition, higher PRS was documented at 5 min post No-nap-13h as compared to at 5 min post Nap-13h (*p* = 0.04 r = 0.56 (large)). However, at 24 h, PRS scores were significantly higher in the Nap-13h condition compared to those at 24 h post Nap-15h (*p* = 0.03; r = 0.59 (large)), (5 min post Nap-15h (*p* = 0.005; r = 0.75 (large)) and 5 min post Nap-13h (*p* = 0.005; r = 0.75 (large)). Furthermore, PRS at 24 h post Nap-15h was higher than at 5 min post Nap-15h (*p* = 0.012; r = 0.67 (large)). Moreover, PRS was higher at 24 h post No-nap-15h than at 5 min post Nap-15h (*p* = 0.017; r = 0.67 (large)), and Nap-13h (*p* = 0.008; r = 0.71 (large)). Similarly, higher values were documented at 24 h post No-nap-13h than at 5 min post Nap-13h (*p* = 0.011; r = 0.68 (large)) and at 5 min after Nap-15h (*p* = 0.015; r = 0.65 (large)), Table 3)

**Table 3:** Delayed onset of muscle soreness (DOMS) and perceived recovery status (PRS) values recorded immediately 5 min and 24 h after the 5msRT.

	Nap-13h		No-Nap-13h		Nap-15h		No-Nap-15h	
	5 min	24 h	5 min	24 h	5 min	24 h	5 min	24 h
DOMS (a.u.)	1.5 (3.25)	1 (2.25)	2 (2)	3 (3)	2.5 (1.25)	3 (2.25)	2.5 (3)	3 (2.25)
PRS (a.u.)	7.50(3.25)	9.00 (1) *^,α^	9.00 (2.00) ^£^	9.00 (1.00) ^&,€^	8.50 (2.00)	9.00 (1.00) ^¥^	10.00 (2) ^#^	9.00 (1.00) ^β^

Values are presented as medians (interquartile range). *: significantly different from 5 min post Nap-13h; ^£^: significantly different from 5 min at nap 13h; ^&^: significantly different from 5 min at Nap-13h; ^#^: significantly different from 5 min at Nap-13h; ^β^: significantly different from 5 min post Nap-13h; ^α^: significantly different from 5 min post Nap-15h; ^€^: significantly different from 5 min after Nap-15h; ^¥^: significantly different from 5 min post Nap-15h, a.u.: arbitrary unit.

## 4. Discussion

The present study explored the impact of 90 min of napping at different times of the day (i.e., 13 h and 15 h) on physical performance, sleep, feeling scale score, perceived exertion, recovery, and muscle soreness in trained youth male athletes. In the current study, naps not only reduced sleepiness and boosted alertness but also produced favorable outcomes for physical performance as well subjective recovery and measures of psychological state. These results confirm our main hypothesis and emphasize the potential benefit of a 90 min nap taken at 13 h on both physical and psychological aspects.

The present study’s findings revealed significant improvements in TD and BD during the Nap-13h condition compared to the other experimental conditions without benefits for FI and PD. These findings support, partially, our hypothesis that early afternoon naps can enhance physical performance after a normal night’s sleep. Such results regarding BD and TD are consistent with those previously reported in other studies that highlighted the beneficial effect of naps on physical performance [3,16,17,19,21,40]. In fact, Boukhris et al. [40] showed that a nap opportunity of 25, 35, and 45 min increased physical performance during the 5mSRT. Similarly, Romdhani et al. [17] showed that both 20 min and 90 min of napping improved repeated sprint performance, with longer naps having stronger enhancing effects on physical performance [17]. Ninety-minute naps could represent a complete sleep cycle and, as a result, could involve the occurrence of quick REM sleep periods [19]. Since REM sleep improves muscular efficiency [41], the improvements of physical performance after napping could be related to REM sleep. Beyond the contribution of REM sleep, it is also important to consider the potential role of slow-wave sleep. This stage is characterized by increased growth hormone secretion, reduced metabolic rate, and enhanced restorative processes, all of which may facilitate muscle recovery and energy replenishment, thereby supporting physical performance and perceived recovery [42,43]. In contrast, REM sleep has been closely linked to motor memory consolidation, emotional regulation, and cognitive processing, which may underlie the observed enhancements in psycho-cognitive outcomes [44]. However, while the lack of significant effects on PD and FI is in line with a previous study using shorter nap durations (i.e., 25, 35, and 45 min) [40], it contradicts the benefits of 90 min naps for FI reported by Boukhris et al. [19], where more than 1 h was allowed for participants after napping. This implies an inability to reproduce the same sprint performance during the 5mSRT. In this regard, it has been reported that FI during the 5mSRT may be influenced by the time between the end of napping and the exercise, indicating that a longer duration between the end of napping and the exercise’s start time may generate a larger decrease in FI [45]. This suggests that sufficient time should be provided to athletes post nap to avoid the negative effect of sleep inertia that appears immediately after waking from sleep. From an energetic pathway, the brief 35 s recovery between repetitions may restrict the athletes’ performance. During data screening, several outcome variables deviated from normality. Accordingly, we prespecified a rank-based, nonparametric primary analysis for those outcomes (see Statistical Analysis Section). To preserve the targeted power when using nonparametric tests, we adjusted the parametric sample-size estimate using the asymptotic relative efficiency (ARE), which quantifies the efficiency of the rank-based test relative to its parametric counterpart under normal theory. Specifically, for repeated measures with k conditions, we used ARE = 0.955 × k/(k + 1) and computed the required nonparametric sample size as n_np_ = n_p_/ARE. Because ARE < 1 under normality, this constitutes a conservative inflation of the parametric estimate to maintain power for the nonparametric analysis which results from elevated anaerobic lactic acid production [46]. The lack of FI and PD under the Nap-13h condition coincided with higher RPE values as compared to the Nap-15h condition. These results are inconsistent with those of previous reports [17,40], in which lower RPE scores were more associated with long naps (i.e., 90 min) than short naps (i.e., 40 min) durations. A potential explanation for this result might be that our participants were not sleep deprived a priori as in the aforementioned studies [47].

The present study revealed that attention measured using the DCT was significantly improved after Nap-13h compared with the control conditions and Nap-15h. These results are in line with those reported in previous investigations reporting an improvement in attention following both 30 min [21] and 90 min of napping [14]. A reduction in sleepiness is among various factors that are positively associated with daytime napping [19]. In the present study, coinciding with the results of DCT, the nap at 13 h led to a reduction in perceived sleepiness, indicating improved alertness, which may partially explain the increase in physical and cognitive performance. In this context, Mantua and Spencer [48] reported that a midday nap reduced sleepiness and improved executive functions. This finding lends credence to the idea that improved cognitive and physical performance was linked to decreased perceptions of sleepiness [19]. Specially, a significant decrease by 54% in sleepiness perception was documented after individuals napped for 90 min [19]. The enhancement of physical and cognitive performance may be linked to the proportion of slow-wave sleep reached during each nap [3,28,40]. A lengthy afternoon nap has been proposed to be similar to a night’s sleep, although it may increase autonomic arousal after waking up more than it does in the morning [49]. Several studies have highlighted the value of the DOMS scale as a practical and accessible tool for coaches and researchers to monitor muscle damage following various types of exercise [22,40]. Its ease of use and non-invasive nature make it particularly useful for tracking recovery and guiding training adjustments in both athletic and clinical settings. However, the current investigation found that DOMS scores were not influenced by napping or by the timing of the nap in response to the 5mSRT. It is possible that the nap had no observable effect simply because there was limited muscle soreness to begin with, reducing the opportunity for a measurable benefit [19]. Such results conflict with what has been recorded by Boukhris et al. [19], who showed that napping had a favorable effect on delayed onset muscle soreness and perceived recovery status, especially, longer naps (90 min). These discrepancies between our findings and those of previous reports may be explained by the quantity of the preceding night’s sleep or the intensity of exercise being performed [50,51]. Since DOMS and PRS were evaluated post nap, the observed results could be related to the severity of sleep inertia following a nap. In fact, sleep inertia is modulated by a number of factors, including prior sleep debt [47], being woken up from slow wave sleep [52], the amount or percentage of slow-wave sleep in a nap [53], and time of day [54]. PRS values recorded at 5 min post nap under Nap-13h and Nap-15h were lower as compared to the control/no-napping conditions. This may confirm an existing effect of sleep inertia after napping. In addition, the lower PRS values after 5 min under the Nap-13h condition may imply a greater amount of slow-wave sleep under this condition as compared to the other conditions. Therefore, the aforementioned homeostatic and circadian processes may interact with subject characteristics (e.g., age, gender, and individual differences in napping experience) to determine the extent of restoration to influence the benefits of daytime napping [47] and may explain the inconsistency across studies.

For psychological state, FS was among the factors that were positively related to napping [17,55]. Regardless of whether athletes were well-rested [40] or sleep-restricted, [17], napping consistently produced beneficial effects on psychological state. In fact, a correlation analysis supports this assumption, where 90 min naps demonstrated positive correlations with changes in vigor, physical performance, and attention [19]. According to Boukhris et al. [40], in well-rested athletes, both 40 min and 90 min naps were associated with reduced tension, depression, fatigue, and total mood disturbance and with enhanced vigor compared to the no-nap condition. However, the present study failed to find a significant impact of 90 min naps on athletes’ affective valence or Hooper index. Since the restorative benefits of napping depend heavily on nap duration, timing, and individual sleep needs, this discrepancy may be explained by variations in s sleep architecture or depth attained during the nap [5,20]. In addition, sleep inertia could be a factor modulating the results from different studies. In particular, the 30 min interval between the nap and the test sessions in this study seems to have been adequate, as participants did not exhibit any signs of decreased readiness or impaired performance in comparison to the no-nap conditions, even though sleep inertia may take up to an hour to dissipate [56].

While the present study provides additive findings to the body of evidence regarding the benefits of napping on physical and cognitive performance, some limitations should be acknowledged. First, due to a lack of equipment, we were unable to perform objective measurements of sleep variables during the 90 min nap opportunities (e.g., actigraphy or polysomnography), making it difficult to confirm whether actual sleep occurred or if simply resting contributed to the observed effects. Additionally, we did not assess the precise duration of sleep inertia or its potential impact on performance, as all test sessions began 30 min after the nap ended. Moreover, the present results are restricted to a small sample of young male athletes and cannot be extrapolated to males of other ages or to female athletes, or to other sports disciplines, which may limit generalizability of the findings. Furthermore, the key feature of the crossover design is that each participant served as his own control and helped control for individual differences, such as baseline levels; carryover and period effects throughout the experiment were not addressed. Beyond these methodological issues, other important limitations relating to the study design should be highlighted. The physical task used was restricted to high-intensity intermittent exercise (i.e., 5mSRT), which may not reflect responses to other forms of exertion. Further research should evaluate the effects of napping on endurance-based activities or short-duration, all-out performance protocols. Another limitation is that only one nap duration (90 min) was tested, so dose–response relationships remain unclear. This is particularly relevant as prior studies suggest that the efficacy of naps may differ based on their duration. Additionally, the unique application of a 90 min nap to all participants did not account for inter-individual differences in sleep needs or chronotype, which could influence the restorative value of napping. Finally, while post-nap activities were standardized in the nap conditions as in the control conditions, exposure to light and TV watching may have influenced the results, although this effect was similar across both conditions. Future research should address these gaps to refine practical recommendations for optimizing nap strategies in diverse athletic contexts.

## 5. Conclusions

This study demonstrated that a 90 min nap at 13:00 was more effective than napping at 15:00 or not napping at all in enhancing some components of physical performance, cognitive function, and perceived recovery in trained youth male athletes. Nappers may conserve a lunch time around 12:00 to 12:30 to benefit from an early afternoon napping (i.e., 13 h) and allow the post-lunch drop in activity to occur naturally. Therefore, from a sports performance perspective, it may be beneficial to provide a nap opportunity of 90 min at around 13:00 before practice or before a high-intensity intermittent exercise to be performed in the afternoon, even if athletes have had a normal night’s sleep. Therefore, practitioners are encouraged to evaluate the efficacy of different nap durations and timings on an individual basis prior to application. Testing nap strategies during training periods can help determine their utility and integration into recovery routines for peak performance. Although muscle soreness was assessed 5 min and 24 h after exercise, this measure reflects perceived muscle soreness rather than the full temporal development of delayed onset muscle soreness (DOMS). Finally, these recommendations should be validated in other populations, particularly female athletes, as well as different ages, sports, and chronotypes, to support more generalizable guidance.

## Figures and Tables

**Table 1:** Total distance (TD), best distance (BD), fatigue index (FI), percentage decrement (PD), and perceived exertion (RPE) recorded during the 5mSRT performed 30 min after a nap at 13:00 or 15:00 or after a no-nap condition.

	Nap-13h	No-Nap-13h	Nap-15h	No-Nap-15h
Best distance (m)	135 (7.5) *^,&^	115 (10)	130 (5) ^&^	125 (10)
Total distance (m)	747.5 (28.75) ^&,#,^*	650.75 (52.50)	732.5 (57.50)	630 (42.50)
Percentage decrement (%)	6.17(6.15)	8.84 (9.78)	3.33 (5.51)	12.67 (11.67)
Fatigue index (%)	9.43 (6.24)	17.86 (10.30)	9.62 (13.41)	15 (12.27)
RPE (a.u.)	5.81/0.89 ^#,^*^,&^	4.54/1.45	4.50/0.67	4.12/0.86

Values are presented as median (interquartile range) and mean/standard deviation, depending on the distribution of each variable. *: significantly different compared to control condition at 15 h; ^&^: significantly different compared to control condition at 13 h; ^#^: significantly different compared to nap at 15 h; a.u.: arbitrary unit.

## Data Availability

The original contributions presented in this study are included in the article material. Further inquiries can be directed to the corresponding authors.

## Supplementary Materials

The following supporting information can be downloaded at: https://www.mdpi.com/article/10.3390/sports13110395/s1, Table S1: CONSORT 2025 checklist.

## Abbreviations

DOMS	Delayed onset muscle recovery
FI	Fatigue index
PD	Percentage decrement
5mSRT	5 m shuttle run test
RPE	Rating of perceived exertion
PRS	Perceived recovery status
DCT	Digit cancelation test
FS	Feeling scale
SSS	Stanford Sleepiness Scale
